# From Empirical Problem-Solving to Theoretical Problem-Finding Perspectives on the Cognitive Sciences

**DOI:** 10.1007/s42113-024-00216-6

**Published:** 2024-10-14

**Authors:** Federico Adolfi, Laura van de Braak, Marieke Woensdregt

**Affiliations:** 1 Ernst Strüngmann Institute for Neuroscience in Cooperation with Max-Planck Society, Frankfurt, Germany; 2 School of Psychological Science, University of Bristol, Bristol, UK; 3 Donders Institute for Brain, Cognition, and Behaviour, Radboud University, Nijmegen, The Netherlands; 4 Language and Computation in Neural Systems, Max Planck Institute for Psycholinguistics, Nijmegen, The Netherlands

**Keywords:** Meta-theory, Problem-finding, Explanation, Cognitive science, Theoretical constraints, Theory development

## Abstract

Meta-theoretical perspectives on the research problems and activities of (cognitive) scientists often emphasize empirical problems and problem-solving as the main aspects that account for scientific progress. While certainly useful to shed light on issues of theory-observation relationships, these conceptual analyses typically begin when empirical problems are already there for researchers to solve. As a result, the role of theoretical problems and problem-finding remain comparatively obscure. How do the scientific problems of Cognitive Science arise, and what do they comprise, empirically and theoretically? Here, we attempt to understand the research activities that lead to adequate explanations through a broader conception of the problems researchers must attend to and how they come about. To this end, we bring theoretical problems and problem-finding out of obscurity to paint a more integrative picture of how these complement empirical problems and problem-solving to advance cognitive science.

*“[...] the quality of the problem that is found is a forerunner of the quality of the solution that is attained, and finding the productive problem may be no less an intellectual achievement than attaining the productive solution [...] A creative solution is the response to a creative problem”* (Getzels, 1979).

## Introduction

How does Cognitive Science make progress? Philosophical analysis of scientific progress has often given a general answer to this question from a problem-solving perspective (e.g., Laudan 1978; Popper 1999). Cognitive Science has a natural affinity with this view, given its foundational focus on the problem-solving capabilities of cognitive systems (e.g., Newell et al. 1958). Likewise, the meta-theory coming from within the cognitive sciences has oftentimes framed the activities of researchers as empirical problem-solving (e.g., Levenstein et al. 2023). While certainly useful to shed light on issues such as theory choice and the role of models in mediating theory and observations, these perspectives begin their journeys more or less when empirical problems are already there for researchers to solve. Therefore, the origin of cognitive-scientific problems, and how their theoretical and empirical components interact, typically remains more mysterious.

How do the scientific problems of Cognitive Science get carved out? And what do they comprise, empirically *and* theoretically? Problem-*finding* is a comparatively obscure topic within cognitive science itself (Getzels, 1979). Similarly, *theoretical* problems (sometimes called “conceptual”; Laudan, 1988) have been given less attention than empirical ones (see Whitt 1988). In this paper, we address this imbalance by attempting to understand the research activities that lead to adequate explanations through a broader conception of the problems researchers must attend to and how they come about. To this end, we bring theoretical problems and problem-finding out of obscurity to paint a more integrative picture of how these complement empirical problems and problem-solving within the broader scientific problems and activities of Cognitive Science. We organize our exploration as follows.

In Section “Categories of Problems and Activities,” we introduce the main types of problems and activities that are to be contrasted but ultimately integrated. We present the conceptual categories of empirical problems, theoretical problems (§“Empirical and Theoretical Problems”), problem-solving, and problem-finding (§“Problem-Finding and Problem-Solving”). We highlight what empirical problem-solving lenses underemphasize and preview a more integrative notion that will guide us throughout.

To bring the theoretical side of scientific problems into focus, it is informative to examine paradigmatic types of theoretical problems; we do this in Section “Plausibility Constraints as Theoretical Problems.” We look at the role of purely theoretical constraints and the theoretical problems that arise in attempts to meet them. Specifically, we describe plausibility constraints as one such problem-generating theoretical device, which we use as a running example throughout.

Theoretical problems can be elusive, and locating problem-finding in the reports and activities of cognitive scientists can be difficult. In Section “Theoretical Problem-Finding Can Be Elusive,” we consider the in-principle discoverability of theoretical problems (§“Discoverability of Theoretical Problems”), how they might elude us in practice (§“How Theoretical Problems Elude Us”), and how this might lead us to construe scientific problems too narrowly (§“Problems, Too Narrowly Construed”).

In Section “Integrating Problem-Solving and Problem-Finding,” we attempt to rebuild an integrative notion of scientific problems for cognitive science that includes theoretical problems and problem-finding. In particular, we describe a broader view of how scientific problems arise (§“The Provenance of Cognitive-Scientific Problems”) and how even phenomena in need of explanation are co-created through theoretical and empirical problem-finding (§“Phenomena Are Co-Created Through Theoretical (and Empirical) Problem-Finding”).

To understand the implications of this broader view, in Section “What We Miss When We Overlook Theoretical Problems,” we consider what we miss when it is absent from scientific and meta-scientific practice. In particular, we consider some consequences of letting empirical problems lead (§“When We Let Empirical Problems Lead”), postponing engagement with theoretical problems (§“When We Postpone Engaging with Theoretical Problems”), and the propensity of small-scale, low-complexity empirical domains to become insulated from theoretical problems (§“The Challenge of Small-Scale Domains”).

Towards the end, we take a look at the positive side of theoretical problems. In Section “Theoretical Problems Can Be Productive,” we describe ways in which they transcend the ordinary use of the word problem, in that they can be productive and carry research forward. This includes the ability of theoretical problems to occasion theory revisions which in turn restructure empirical problems (§“Theory Revision Driven by Cognitive Scope Violations”) and their ability to guide the exploration of the boundaries of plausible explanations (§“Exploring the Boundaries of Plausible Theories”).

Finally, in Section “Problem-Finding Without Problem-Solving,” we briefly draw attention to the fact that, by necessity, problem-finding must often be conducted even without problem-solving. Section “Outroduction” closes with a few overarching remarks as we consider what this integrative view means for cognitive science more broadly.

Throughout this journey from empirical problem-solving to theoretical problem-finding perspectives, we will encounter two overarching themes: (1) from a scientific point of view, we can only hope to arrive at adequate explanations if we consider empirical and theoretical problems on equal footing, and (2) from a meta-scientific perspective, we can only account for how scientific problems arise and how adequate explanations are discovered if we consider both empirical and theoretical problem-finding/solving together.

With this road map in place, let us begin.

## Categories of Problems and Activities

To begin our exploration, we first draw necessary distinctions between empirical and theoretical problems as understood in the philosophy of science and between problem-solving and problem-finding—a much less explored contrast.

### Empirical and Theoretical Problems

The idea of cognitive science as empirical problem-solving can be a powerful organizing thought (e.g., Levenstein et al. 2023). It can help us zoom in on the local purposes of theories and models, and how they are used in practice. Yet, the modal reading of this analogy is often through an empiricist lens that has been argued to hold back cognitive science more generally (see Goldrick 2022; van Rooij and Baggio 2021, 2020).

Radical empiricist views of science hold that it is primarily the accumulation of rigorously gathered observations and the detection of regularities therein which give rise to and form the bedrock of theories, and that the latter should be evaluated primarily on how precisely they retrodict,^1^ those observations, predict new ones, and lead to better control of systems of interest (e.g., Nosek et al. 2018). In other words, on this view, theories emerge from empirical problem-finding/solving activities and are appraised on how successful they are at solving empirical problems,,^2^

Interestingly, it was to counter these empiricist lenses that it was deemed necessary to foreground a distinction between empirical and non-empirical problems (Laudan, 1988; Whitt, 1988 and refs. therein). The bipartite account of scientific problem-solving was framed as follows. A puzzling phenomenon requires an explanation and hence poses an empirical problem. A scientist interested in this phenomenon may put forth a theory in an attempt to explain it (i.e., “solve” the empirical problem). In addition to retrodicting observations associated with the phenomenon, the theory might make other predictions. If these are contradicted by subsequent observations, the latter become anomalies posing further empirical problems. However, the theory itself might also conflict with other accepted theories or principles. For instance, a computational cognitive theory might run against accepted principles in the Theory of Computability and Complexity (see Garey and Johnson 1979; Reiter and Johnson 2013). These clashes represent non-empirical problems (sometimes called *conceptual* problems) that need to be resolved. From here onward, we will refer to problems of this non-empirical kind as *theoretical problems*.^3^

The importance of introducing these distinctions was, among other things, that it allowed philosophers to frame the following meta-theoretical observation: it is often the case that a new theory manages to account for so-far-unexplained empirical observations only at the cost of introducing a number of theoretical problems (Laudan, 1988). Furthermore, it was necessary to point out that *“[...] it is possible that a change from an empirically well-supported theory to a less well-supported one could be progressive, provided that the latter resolved significant conceptual difficulties confronting the former”* (*ibid.)*.

Even when a certain domain of inquiry is initially construed around an empirical phenomenon, the issues quickly revolve around theoretical problems (Popper, 1999). There are fewer ways of accounting for observations while remaining self-consistent and cohering with existing knowledge than without these constraints; therefore, a substantial portion of time and effort is devoted to engagement with these theoretical problems. However, the pragmatic view of science as problem-solving tends to focus disproportionately on *empirical* problem-solving (e.g., Nosek et al. 2018; Yarkoni and Westfall 2017). This is despite the overarching goal of cognitive science of accounting for behavioral observations *subject to cognitive-theoretic constraints*. More generally, resistance to admit theoretical problems and their appraisal into accounts of how scientists arrive at explanations seems to have been common beyond cognitive science. *“[T]he usual response when confronted with cases in which theories are being appraised along non-empirical vectors has been to deplore the intrusion of these nonscientific considerations [...]”* (Laudan, 1988).

**Fig. 1:**
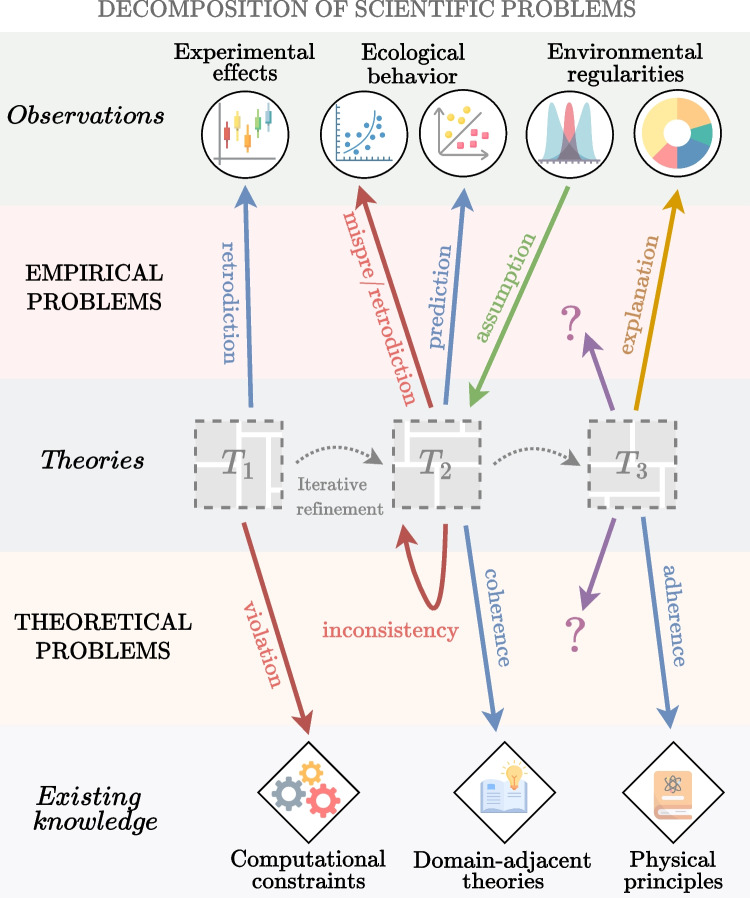
Schematic of some of the possible relationships that can exist between successively conjectured theories, pre-existing knowledge, and observations, such that empirical and theoretical problems give rise to scientific problems. *Empirical problems* (top center) are located between theory and observation, while *theoretical problems* (bottom center) are located between theory and existing knowledge. Red arrows indicate relationships that play a role in *problem-finding*, while blue ones are associated with *problem-solving*. Other relationships between theory and observation that are often neglected by empirical problem-solving accounts include assumption (green arrow) and explanation (golden arrow) of empirical observations. In the process of iteratively refining or comprehensively updating theories to solve problems (center; left to right), one or more of these relationships can change or become unknown (purple arrows). During this adjustment, a substantial reconfiguration of the space of empirical/theoretical problems can occur as certain *observations*—and, less frequently, some *existing knowledge*—might go from relevant to irrelevant and vice versa

### Problem-Finding and Problem-Solving

*“[...] to call attention to the neglect of problem-finding as against problem-solving in cognitive science”* (Getzels, 1979).

The discussions alluded to thus far, regarding empirical versus theoretical problems, have often been too narrowly framed in that they revolve mainly around the idea of problem-*solving*. Curiously absent is how problems (let alone *theoretical* ones) are found in the first place: the process of problem-*finding*. The tendency to focus on problem-solving at the expense of problem-finding can be gleaned in the very foundations of Cognitive Science (e.g., Newell et al. 1958), where the pinnacle of human cognition as an object of study was deemed to be its problem-solving capabilities (see also Getzels 1979). Similarly, in research reports, cognitive scientists are more likely to discuss how empirical phenomena (empirical problems) are predicted (solved), less so how empirical or theoretical problems are found or how the latter are dealt with (see also Newell 1973). This is striking considering that even (so-called empirical) phenomena can be the outcome of sophisticated, perspectival theoretical processes (Massimi, 2022).^4^

To preview a more integrative view, *scientific problems* arguably arise through the interaction between theoretical and empirical problems (see Fig. 1). *Theoretical problem-finding* is the heterogeneous set of processes through which theoretical devices (e.g., mathematical and computational models, qualitative accounts, conceptual analyses) are explicitly made to confront other existing knowledge (e.g., domain-adjacent theories, computational constraints) in order to remove flaws of various kinds in conjectured theories (Fig. 1, bottom). This is in contrast to *empirical problem-finding*, where phenomena of interest are framed as explananda—against the backdrop of theories and theoretical problems—and critical data are sought such that theory can be confronted with observation, for instance, to compare the retro/predictions of candidate theories or to point to deficiencies in established theories (Fig. 1, top).

The reason theoretical problem-finding is largely absent from our accounts of how cognitive scientists make progress seems to be an overly narrow and compartmentalized conception of scientific progress. In particular, one that centers an impoverished notion of *empirical* problem-*solving*: when models retrodict observational/experimental data. As we move forward, we will attempt to rebuild an integrative view and put the problems faced by cognitive scientists back together. One of our main points will be that it is not possible to frame so-called empirical problems in meaningful ways in the absence of relevant theoretical problems. Let us first briefly describe theoretical problem types that will serve as running examples later on.

## Plausibility Constraints as Theoretical Problems

*“[...] of course, science is about finding out what is actual. But you should care about what is possible because, in the absence of a God’s-eye access to reality, knowing what is possible is an important (dare I say, it is the only) guide to find out what is actual”* (Massimi, 2022).

In order to make the core issues we introduced above more concrete, we will occasionally delve into illustrative examples. These include hypothetical scenarios and real instances of theories running into specific kinds of theoretical problems. These, we argue, are caused by theoretical plausibility constraints. Two example constraints featured in this paper are *tractability* (van Rooij, 2008; van Rooij et al., 2019) and *cognitive scope*. We briefly explain the rationale behind plausibility constraints before moving on.

Humans, as well as all other natural and artificial cognitive systems, have limited resources available for computation. This limitation imposes a constraint on the set of cognitive capacities that can be plausibly conjectured. That is, plausible theories of cognition cannot (ultimately) put forward computations for which there cannot exist resource-efficient procedures. This, in a nutshell, is the *tractability* constraint on cognitive theories (for details on how this intuitive notion was shaped and variously formalized, see van Rooij 2008; Wareham 1996). We cannot apprehend cognitive capacities directly, but sophisticated mathematical machinery exists to make these aspects of our theories intelligible to us. *“[V]aluable insights can be derived via complexity-theoretic hardness results which show what cannot be done over a wide range of machines and input sizes”* (Wareham, 1996). As another example of plausibility constraint, consider *computability* as a lower bar (Fleck, 2009). Uncomputable functions, those for which there cannot exist procedures to compute them (let alone efficient ones), cannot be part of plausible cognitive theories.

Consider next another plausibility constraint: *cognitive scope*. A theory violates a *cognitive scope* constraint when it incorporates an assumption that is at odds with the real-world generality of the cognitive capacity studied. The violation of *cognitive scope* often stems from adopting an assumption that is unnecessary theoretically but crucial empirically (i.e., to “solve” the problem; we will show how this can happen in §“Theory Revision Driven by Cognitive Scope Violations”). As a result of this, the local conceptualization and modeling of the capacity underestimates human cognition. In this sense, the (implicit) theory represents an *undergeneralization*.

These are just examples of some problem-inducing constraints which happen to have broad applicability. Related constraints under the general “plausibility” umbrella are *evolvability* (Barron et al., 2023; Brown, 2014; Kaznatcheev, 2019; Rich et al., 2020), *learnability* (Angluin, 1992), and *developability* (Abouheif et al., 2014; Laland et al., 2015), among others (see Blokpoel 2018, for a similar overview of theoretical constraints on explanations of cognitive capacities). Many other types exist, of various levels of generality and applicability (Adolfi, 2024).

Three observations will be useful to bear in mind going forward: (1) constraints such as *tractability*, *computability*, and *cognitive scope* represent a priori explanatory challenges and hence can shape both theoretical and empirical aspects of scientific questions; (2) these constraints can generate theoretical problems when confronted with existing theories and hence prompt a reassessment of what the relevant empirical problems should be; and (3) explanations involving violations of these constraints can be rejected or amended, partially or completely, on purely theoretical grounds. We will elaborate gradually on each of these as we progress through the rest of the sections.

## Theoretical Problem-Finding Can Be Elusive

Theoretical problems, as a conceptual category, have been important to understand how researchers arrive at better explanations (see Whitt 1988, and refs. therein). However, our tradition of research reporting is not always transparent on this, and readers of the cognitive science literature could not be blamed for underestimating the role of theoretical problem-finding. It is possible to attribute the (in)visibility of theoretical problems and problem-finding to various qualitatively different causes. We explore some of them next.

### Discoverability of Theoretical Problems

From a meta-theoretical standpoint, not all problems are readily discoverable from just any of the various forms of theory. For a given theoretical problem to be discoverable, theoretical objects must be available that are amenable to scrutiny in problem-type-specific ways. Some kinds of theoretical objects (e.g. verbal statements, varieties of computational models, and mathematical descriptions) will lend themselves to some analyses but not others. For some problems to be uncovered, a prerequisite might be to have computational models (see Guest and Martin 2021; van Rooij 2022) to perform theoretically guided simulations. Other kinds of problems are only visible through modeling at a more abstract level that enables broadly generalizable analyses. For instance, uncovering the sources of intractability in a cognitive theory requires a formulation at the computational level of analysis (Marr, 1982; Varma, 2014; van Rooij et al., 2019) which is usually not available or easily obtainable from verbal, or even algorithmic, theories (see Adolfi et al. 2022; Woensdregt et al. 2021). Similarly, discovering (possibly mistaken) philosophical assumptions can be harder to do from formal models alone than from theories that make their conceptual commitments explicit. Quantitative theories afford critiques that qualitative ones do not, and vice versa. Each possible theoretical problem might require a certain “view”—or indeed, a new development—of the theory at the appropriate level of detail, idealization and abstraction.^5^ In the absence of this view, theoretical problems remain out of sight and out of mind.

From a sociology of science perspective, “it is often outsiders who see a problem first” (Popper, 1999). Specifically, these problems are often better spotted from the point of view of a theorist who is also a meta-theorist. Since theoretical problems often come from this “outsider” perspective (see Guest, 2024, for related issues), they might take longer to be assimilated into the mainstream research strands where theory and experiment meet.

### How Theoretical Problems Elude Us

Perhaps due to (i) the loose connection that often exists between experimental and theoretical strands in any given research domain, and (ii) a tradition of research reporting centered around empirical data, theoretical problems and problem-finding can easily elude an honest reader of the literature in the cognitive sciences. For instance, it is often the case that the focus of reports is mainly on what an experimental procedure or computational model can do in terms of data generation or fitting and the patterns that can be gleaned therein. That is, they mainly revolve around empirical problem-solving, narrowly construed. Much less common is to focus on how these modeling and analytic devices might stem from having tackled theoretical problems and how these fit within the broader scientific questions.

For a reader, this might have the undesirable effect of giving the appearance that theoretical problems are not at play and that theoretical problem-finding has not taken place. But as a scientific custom, it also has another unintended consequence: it keeps theoretical problems at a safe distance from so-called empirical problems. When this happens, necessary constraints that would otherwise cause theoretical problems might be implicitly and unintentionally assumed to hold. These (often implicit) assumptions are seldom checked (Adolfi et al., 2022; van Rooij et al., 2008). Concerns about such theoretical constraints are often downgraded to informal discussion of the underlying theory or framework. Often they are relegated to the choice of language or framework to express our theories, a choice that is often justified in practical terms or alluding to technological concerns rather than theoretical ones.

It is tempting to dismiss theoretical problems altogether as they arise on the grounds that they do not make a difference in practice. From a pragmatic point of view, it is possible to cast theoretical principles—in particular, those that would otherwise cause theoretical problems—as “non-falsifiable” or “non-testable,” therefore not within the purview of efforts to solve empirical problems. In certain scenarios, theoretical problems can be understood in this way as unrelated to what is the case in practice. This is possible because it is often the case that even when a theoretical problem is brought to the foreground, it does not change empirical predictions (*for the observations at hand*.)

If not dismissing, then postponing can often seem like a reasonable course of action. Using an operationist lens, one could argue for putting off a given theoretical problem until such time as it might be embodied in datasets and empirical tests (e.g., see Yarkoni and Westfall 2017). For instance, this rationale is embedded in the research culture of many fields drawing heavily from empirical machine learning frameworks. For any given problem, it materializes only when a “benchmark dataset” is constructed to support model evaluation (see Bender and Koller 2020; Birhane et al. 2022; Raji et al. 2021).^6^

### Problems, Too Narrowly Construed

*“[...] in real life, many exercises in which model choice relies too heavily on quantitative measures of performance are essentially selecting models based on their ancillary assumptions. It is unclear to me if this solves a scientific problem of interest”* (Navarro, 2019).

From a pragmatic perspective, it would seem rational to substitute relevant empirical problems for theoretical ones whenever these arise. Of relevance here is how this strategy could contribute to a narrow construal of the scientific problems cognitive scientists face.

This pragmatic facet of research strategizing appears in various forms in many domains. The following kind of appraisal (selected for no particular reason) is not rare and can be easily misinterpreted as shallow empirical problem-solving: *“[...] the question of whether [large language models] can inform our theories of human language understanding is first and foremost an empirical question”* (Pavlick, 2023). There are reasonable interpretations of these kinds of statements that do not necessarily contradict an integrative view of theoretical development and empirical observation. The issue with construing scientific problems narrowly around empirical problem-solving, however, is that one can inadvertently remove (from view) the challenge of finding and solving the theoretical problems that are inextricably linked (i.e., the lower half of the schematic in Fig. 1). For instance, on the status of large language models as theoretical devices that might inform questions of human cognition, it might seem sensible to impose a purely behavioral/neural criterion (e.g., Schrimpf et al. 2020). That is, from a pragmatic point of view, we might want to substitute the original question with whether an empirical test fails to show a difference between humans and models. If it fails, then models are similar enough that they should be informative. However, there are innumerable ways in which we, as researchers, can be sidetracked by deploying this kind of methodological rule in isolation (see Bowers et al. 2022, and refs. therein). Models can mimic human behavior and neural patterns through different underlying mechanisms, and only a select few of them are generally of scientific interest (Guest & Martin, 2023). A safer methodological rule would also include, for instance, an appraisal of mechanistic explanations of how both human language and large language models work (e.g., Adolfi et al. 2023; Oota et al. 2023), and how each of these accounts copes with relevant theoretical problems. These may include, for example, considerations on the computational expressivity of the transformer architecture of language models (Strobl et al., 2023) as compared to human language capacities.

More generally, radically pragmatic methodological rules for cognitive science have been criticized for their tendency to reframe scientific questions such that they become approachable with bottom-up quasi-mechanical procedures, possibly reflecting misconceptions about how knowledge can plausibly be produced. These include, to give some examples, (a) that cognitive explanations are mainly obtained by the discovery and accumulation of stable experimental effects, emphasizing empirical testing (see van Rooij and Baggio 2021; b) that much of cognitive science is an amalgam of automatable tasks (see Adolfi and van Rooij 2023; Rich et al. 2021; c) that cumulative cognitive science means conducting massive (atheoretical) replication studies (see Devezer and Buzbas 2021; d) that scientific exploration can be proceduralized (see Devezer 2023; e) that theory appraisal can be reduced to model selection through predictivity rankings (see Bowers et al. 2023; Guest and Martin 2023); and (f) that implementation-first approaches emphasizing neural data have a claim to primacy over approaches emphasizing functional analysis (see Niv 2021; Poeppel and Adolfi 2020). These pragmatic strategies appear elsewhere in cognitive science as well, for example, in statistical modeling: *“ [...] much of the model selection literature places too much emphasis on the statistical issues of model choice and too little on the scientific questions to which they attach”* (Navarro, 2019). In other words, there seems to be an ever-present risk that in the pragmatic substitution of empirical questions for the inevitably intertwined empirical-theoretical problems, we end up impoverishing our original scientific questions.

## Integrating Problem-Solving and Problem-Finding

*“Need problems be found? [...] The world is of course teeming with dilemmas. But the dilemmas do not present themselves automatically as problems capable of [...] even sensible contemplation. They must be posed [...] in fruitful [...] ways if they are to be moved toward solution. The way the problem is posed is the way the dilemma will be resolved*” (Getzels, 1979).

We have been arguing that in conceptualizing cognitive science within the frame of problem-solving, we have generally favored a skewed view of scientific problems. Empirical problem-solving is often centered at the expense of theoretical problem-finding due to an overly narrow conception of what scientific problems comprise and what solving them entails. The result is that we lose sight of how empirical and theoretical aspects of scientific problems influence one another. We shall now gradually bring back into focus a more integrative view of scientific problems which deobfuscates how theoretical problem-finding, together with empirical problem-finding, gives rise to scientific problems of interest in cognitive science.

### The Provenance of Cognitive-Scientific Problems

*“In order to probe this question, ideas that fit current knowledge as well as possible must be formulated”* (Barlow, 1972)

Accounts of how Cognitive Science makes progress usually begin with empirical problems that need solving. These, the story goes, are embodied in problematizing datasets whose patterns need to be accounted for. For instance, a dataset of neural activity recorded while primates are presented with images might in some sense capture the problem of how the brain computes visual representations that are useful to behave appropriately (e.g., Schrimpf et al. 2020). But where do these problems come from? That usually seems less clear and of comparatively little importance. Two issues with the empirical problem-solving frame stand out. Firstly, the word empirical (in opposition of theoretical or conceptual) may give an impression of autonomy of empirical problems that these do not possess with respect to theoretical problems (see Fig. 1; as theoretical [empirical] problems are dealt with by successive theory variants, empirical [theoretical] problems may revert to unsolved or their status and/or relevance might become unknown). And secondly, the disproportionate focus on problem-solving leaves little room for the issues of problem-finding: how problems arise in the first place. Scientific problems are not given. They need to be searched for, actively created or carved out. We invest in particular kinds of scientific activity in order to do this.

All problems of scientific interest arguably arise due to gaps, flaws, and conflicts in and between our explanations. When we puzzle over, for instance, how it is possible for people to communicate efficiently and resolve misunderstandings (see van Arkel et al. 2020; van de Braak et al. 2021), it is not because we are awestruck by intrinsically mysterious, unexpected, or extraordinary observations. Human communication is a wholly unsurprising, commonplace thing. Yet, when looked at through the lens of existing, fallible explanations, communication can (rightly) acquire a problematic appearance (see Micklos and Woensdregt 2022). This is because our cognitive explanations of how humans do such things as resolve misunderstandings contain large gaps (we go into case studies in sections §“The Challenge of Small-Scale Domains” and §“Theory Revision Driven by Cognitive Scope Violations”). And indeed researchers invest time in locating and delimiting the source of these theoretical problems (e.g., van de Braak et al. 2021; Woensdregt et al. 2021). It is through these interlocking cycles of observation and theoretical activity that the capacity for communication is carved out as a scientific problem for cognitive science at all (see van Rooij and Baggio 2021). That a problematizing dataset can at some point be construed as embodying the “empirical problem” of misunderstanding resolution is, in this scheme, a much more parochial affair than the broader scientific problem of explaining human communication.

The fundamental point is that problems do not reveal themselves ready for us to solve. We seek them out and shape them, not only “out there” but also “in here,” within our own explanations.

### Phenomena Are Co-Created Through Theoretical (and Empirical) Problem-Finding

*“Traditionally scientists are said to explain phenomena that they discover in nature. I say that often they create the phenomena which then become the centerpieces of theory”* (Hacking, 1983).

The quote above highlights a decades-old conceptual flip that emphasized the until-then-neglected role of experiments. Here, we will argue for a similar conceptual flip but with a focus on how theoretical problem-finding contributes to the creation of phenomena qua primary or secondary explananda. This angle on the issues emphasizes the role of “*perspectival modeling rather than experiments in delivering phenomena*” (Massimi, 2022).^7^ On the view we are emphasizing here, empirical problems are but one epistemic tool to bring already existing ideas in conflict with each other. It is only in this sense that “they” may be said to provide scientific problems for cognitive scientists. Arguably, researchers actively carve out empirical phenomena by bringing to bear theoretical problems.

Let us get preliminaries out of the way before delving into this further. We adopt a distinction between primary and secondary explananda (Cummins, 2010; van Rooij & Baggio, 2021). Cognitive capacities (e.g., the ability to communicate, to reason about sensory experiences) are the primary things to explain in Cognitive Science, by definition. Other phenomena, such as experimental effects (e.g., the face inversion effect, the Stroop effect), are certainly of interest but as secondary explananda. That is, cognitive science, except perhaps in contrived scenarios, is not generally interested in, for instance, the experimental face inversion effect except in the context of explaining how face perception—and visual perception more broadly—works in the real world.

Attempts at investigating cognitive capacities, on the one hand, can foreground explanatory flaws in various ways. For instance, this can happen when it proves challenging to explain them while adhering to cognitive-theoretic plausibility constraints (e.g., tractability). Similarly, these flaws can emerge from our efforts to integrate the explanation of cognitive capacities with other cognitive explanations (known as *coherence* in the philosophy of science; see Douglas 2013; Keas 2018). Experimental effects, on the other hand, might be the focus of model comparison because they foreground a clash between a priori plausible theories or because they highlight a flaw in an established theory that fails to explain them. An explanation of effects can also be of interest for other (e.g., applied) goals. In any case, problematizing phenomena become such, in no small part, by virtue of providing avenues to foreground theoretical problems. Conversely, bringing theoretical problems to the foreground allows us to clarify what the relevant problematizing datasets (empirical problems, to entertain this terminology) might be. This highlights how the space of empirical and theoretical problems can reconfigure each other as theories are revised (see Fig. 1).

To elaborate on this last point, consider that under a fallibilist epistemology (e.g., Deutsch 2012), all (so-called empirical) problems, in the sense of phenomena in need of explanation, are apparent problems. As we have seen, this problematic appearance is a function of existing, inevitably fallible explanations that act as lenses for observing and conceptualizing cognitive phenomena. That is, flaws in available explanations might make it so that certain observations acquire a problematic appearance. A problematic appearance is thus always due to a theoretical misconception in the observer. We just need to find it.

The following is a brief case in point (see Adolfi et al. 2022, for details) to illustrate how theoretical problem-finding and solving can reconfigure the space of phenomena and hence of empirical problems (see Fig. 1). In many cognitive domains where segmentation processes are involved (e.g., speech recognition, event processing, action parsing, music perception), researchers have wondered whether certain observed regularities in the environment might be leveraged by cognitive systems to perform efficient computations. The focus on these empirical patterns was motivated by the need to discover constraints on the segmentation subcomputation, otherwise believed to be an intractable task. Several empirical research programs were launched to characterize these regularities and their interactions. However, the efforts around, and interpretation of, these specific empirical phenomena were due to an informal theoretical assumption that had gone unexamined. It involved a possible misconception about the hardness of the segmentation computation and the possible role of such empirical regularities in alleviating it (Adolfi et al., 2022). Once this possibly misconceived assumption is removed, the original role for the regularities in existing cognitive explanations can vanish. The relevance of these observations as problematizing datasets can thus be reconsidered, and the space of empirical problems can be reconfigured (*ibid.*).

The ongoing discussion should suggest that phenomena in need of explanation are actively carved out by cognitive scientists when they observe cognitive behavior, or isolate secondary empirical regularities, *through the lens of existing explanations and theoretical constraints*. Much in the same way that theories and theoretical problems can be “empirical problem”-laden (Levenstein et al., 2023), in the sense that they arise from and depend on efforts to explain particular observations, empirical problem-finding is theory-laden. In particular, this lack of automaticity or autonomy of empirical problem-finding highlights the active role of researchers in bringing to bear theoretical problems to carve out phenomena as scientific problems. *“[E]ven if scientists have a hypothesis about what model to use for a particular investigation, how do they apply the model to the world? More specifically, what exactly do they apply the model to? [...] Merely stating that they applied the model [...] does not do justice to the time and effort spent ‘preparing’ the ‘world’ so that the model could be applied to it”* (Elliott-Graves, 2020).

## What We Miss When We Overlook Theoretical Problems

A lot can go wrong if we neglect or postpone thinking in terms of theoretical problems. In this section, we explore some of the ways in which we can be misled, get stuck, waste time and resources, or otherwise miss opportunities to improve our knowledge of cognitive systems.

### When We Let Empirical Problems Lead

*“[...] empirical adequacy is a poor starting point that could have us picking from among the wrong theories in many contexts.”* (Bhakthavatsalam & Cartwright, 2017).

Failing to account for empirical observations and failing to attend to theoretical problems have different consequences. When we fail to account for data, we are failing to pre/retrodict aspects of the system of interest. Our ability to evaluate our understanding of the cognitive system, however, remains intact. That is, we can still keep track of the state of our theories, their virtues, and flaws. On the other hand, striving to account for more data while ignoring theoretical problems directly distorts the artifacts that mediate our understanding. This is because it is always possible to accommodate observations in innumerable ways. However, most of these accommodations will generate conflicts with existing, possibly well-established knowledge. If these clashes are systematically overlooked, this amounts to giving up our ability to remove errors that make our theories implausible as explanation-generating devices. These theoretical devices hence lose force as thinking and observation tools. This loss is due to the increasing likelihood that statements derived from the theory, including empirical predictions and conjectures about what data is relevant at all, are the consequence of its overlooked flaws. Sacrificing theoretical constraints distorts our reading of empirical phenomena (e.g., cognitive system behavior)^8^ and diminishes our ability to assess the strengths and weaknesses of our explanations.

### When We Postpone Engaging with Theoretical Problems

From an empirical problem-solving mindset, it would seem that we can set up the following procedure to arrive at good theories: (1) take a theory or model that does reasonably well at pre/retrodicting as much empirical data as possible, never mind its theoretical merits for now, and then (2) iteratively fix its theoretical gaps via criticism (without sacrificing empirical adequacy or accuracy) until we arrive at a theory which is difficult to improve further while meeting all relevant criteria. This and related commonsense ideas are unlikely to work in practice. The reason is that the process of producing these good-for-now, hard-to-improve theories cannot be proceduralized efficiently at all, let alone as just described. The landscape of possible explanations is provably far too hard to navigate—both from the functional (Rich et al., 2021) and the implementational perspective (Adolfi & van Rooij, 2023)—to expect that adjusting one constraint at a time can be a sure route to good theories. Consider one salient implication. For any given constraint-violating theory, it is not at all clear that a path to better theories exists such that all intermediate theories violate fewer constraints. The main takeaway should be that leaving theoretical problem-finding as an afterthought of empirical research activities will likely get us stuck.

### The Challenge of Small-Scale Domains

*“...if we only solve simple problems, we may never learn how to think about the complex ones”* (Navarro, 2019).

The natural phenomena that cognitive scientists try to explain often take place on a large scale. This scale can be expressed in terms of the spatio-temporal extent, diversity, or complexity of the input to the cognitive process of interest, or of the stored knowledge that is used in this process, among others. Empirical studies aiming to capture such processes necessarily simplify, idealize, and abstract away from some of the breadth and complexity of the real-world phenomenon (see Potochnik 2020). That is, experiments usually take place on a small, and possibly also low-complexity, scale. The same is often true for computational models which aim to mediate between theory and experiment. This practical simplification, however, can come, often implicitly, at great cost to theory and explanation.

Here, we illustrate the challenges to theory caused by plausibility constraints not being apparent due to small-scale domains. We discuss a case where theory development progressed mainly through small-scale computational models and experiments, and we bring the consequences into focus. We will see how a broad class of computational models and explanations of natural language properties harboured a detrimental theoretical problem. Furthermore, we will see how theoretical problem-finding uncovered it by proving a violation of plausibility constraints, tracing the source to a particular component of the models (for details, see Woensdregt et al. 2021), and locating the broader issue in a disproportionate focus on small-scale domains. But first, we give some background on the phenomenon of interest, the embedding cognitive domains, and the computational models therein.

The phenomenon of interest is language and its structural properties, in particular, those properties shared by most or all natural languages. A classic example of such a *design feature* (Hockett, 1960; Pleyer & Zhang, 2022; Wacewicz & Żywiczyński, 2015) that we find in all human languages is *compositionality*: that the meaning of a sentence is (most often) made up of the meaning of its parts *and* the way in which those parts are combined (Martin & Baggio, 2020; Partee, 1984; Pylkkänen, 2020; Woensdregt et al., 2024). Given that language is a cultural artifact that lives in and is produced by the minds of humans, researchers have looked for cognitive explanations for these structural features of language. Are there particular (cognitive) constraints or pressures on the way language is used, learned, and passed on over generations that could explain why different languages across the world, from many different language families, share the same structural properties? (Chater & Christiansen, 2010; Christiansen & Chater, 2008; Kirby, 2017; Smith, 2014, 2018; Spike, 2018; Tamariz, 2017; Tamariz & Kirby, 2016). For example, the property of compositionality may be explained as a result of a trade-off between a need for expressivity (wanting to be able to communicate many different meanings) and a need for compressibility (which makes the language learnable and generalizable; Kirby et al. 2015; Motamedi et al. 2019; Raviv et al. 2019). This is just one illustrative example of the kinds of explanations that have been investigated using the class of agent-based models we focus on here.

Agent-based modeling plays a major role in developing these kinds of explanations because these theories involve interactions between different levels of organization (individual vs. population) and different timescales (from conversation to language acquisition, to cultural evolution). Computational models allow researchers to specify their theories (Guest & Martin, 2021) and explore the dynamics that ensue (Madsen et al., 2019). In the class of agent-based models, we focus on here—*iterated Bayesian language learning* models—the cultural evolution of language is simulated by having agents in a population communicate with each other, and having new generations of agents enter the population and learn a language by observing the communicative behavior of agents from the previous generation (see Ferdinand, 2024; Kirby et al. 2014; Woensdregt et al. 2021, and references therein). This allows researchers to gain insight into the dynamics that would follow from a particular theory, by manipulating various aspects of the computational model and comparing the results of computer simulations under different conditions.

Over the past $\sim$15 years, this computational modeling work has been complemented with experimental research, in which the process of cultural evolution of language is simulated in the lab. In these iterated learning experiments, transmission chains are created by having human participants learn a miniature (i.e., small-scale, low-complexity) artificial language, followed by a communication phase in which they are asked to use the language in pairs, after which a next pair of participants (simulating the next “generation” of learners) is trained on the output of the previous participants, and so on (see Tamariz, 2017; Tamariz & Papa 2023), for a reviews).

In many papers published in this field, it is shown that under the conditions that the theory carves out as important, the (small-scale, low-complexity) languages that result at the end of the iterated learning chain show a similar property to that of naturally occurring languages that the theory aims to explain (e.g., compositional structure). However, the exact cognitive biases or communication strategies of the human participants are hard to ascertain. Therefore, these experiments are often combined with computational modeling work that directly implements and manipulates the factors of import put forward by the theory. When the simulation results (also small-scale, low-complexity) obtained with such a model show a similar pattern to the results of the behavioral experiments, this is considered corroborative evidence that the theory indeed explains not only the outcome of the small-scale experiment but also the real-world pattern observed in the world’s natural languages on the ecological scale.

However, using computational complexity analyses (§“Plausibility Constraints as Theoretical Problems”), Woensdregt et al. (2021) showed that the learning model at the core of a subset of these computational models^9^ violates a core plausibility constraint: they are formally intractable. That is, this particular class of models cannot in principle be scaled up from the small-scale domain (usually not exceeding lexicon sizes of 4 words and 4 meanings) to the ecological scale (adult native speakers of English know $\sim$25,000–50,000 words; Brysbaert et al. 2016). This property is intrinsic to the theories and models (i.e., it cannot be mitigated by better procedures or technologies). The intractability could be traced to the use of Bayesian inference as the model of learning, in which learners infer a language from the communicative behavior they have observed, by considering the entire hypothesis space of logically possible languages and computing the likelihood that each of those languages would have produced the observed data. If the model of language that is used is a lexicon of signal-meaning mappings, the space of logically possible languages grows exponentially as the number of signals and meanings is increased, and this space cannot be searched efficiently (see Woensdregt et al. 2021, for more details). On a practical level, this intractability finding proves that simulations run with this particular class of models fundamentally cannot go beyond a small scale. Furthermore, the formal results have important theoretical consequences: (i) we cannot take it on faith that the small-scale effects would scale to large, real-world lexicons, and (ii) even if the large-scale effects would exist, the theory as implemented in the computational model would not explain them.

The practical consequence of this intractability was probably intuited to some extent by the researchers implementing these computational models, as several articles mention mathematical tricks or approximation methods to bring down simulation run times. This can be a useful practical aid, but the main insight for our purposes is that it reveals a problem-*solving* mindset that works against theory and explanation. It treats the issue as an engineering problem, whereas Woensdregt et al. (2021) showed it is a fundamentally theoretical problem. What was necessary for theoretical progress (in this case) was formal analyses, which, as we will see in the next sections, allow us not just to identify what makes theories implausible but also provide ways of sculpting them into more plausible ones.

## Theoretical Problems Can Be Productive

*“[...] as an explanation, it has serious problems. Problems are fruitful things [...]”* (Marletto, 2021).

Not everything that theoretical problems bring is bad news. Theoretical problem-finding can be crucial even to solving ostensibly empirical problems. *“[T]here are at least three ways in which a theory may undergo conceptual growth and refinement, and thereby enhance the conceptual resources which it supplies for empirical problem-solving [...] through the fine-tuning of its concepts; through the achievement of greater consilience; and through the appropriation of the conceptual resources of theories in other domains”* (Whitt, 1988). This is because, as can happen with empirical problem-finding, theoretical problem-finding may allow us to pinpoint where our understanding is lacking and to explore plausible ways to improve it. But for this, we must meet the challenge of cognitive-theoretic constraints head on. Next, let us take a look at how this can bring a theory out of a state of entrenched misconceptions and thus improve our understanding and point the way forward.

### Theory Revision Driven by Cognitive Scope Violations

Experimental setups, often embodying small-scale, localized, or low-complexity versions of real-world phenomena, necessarily introduce ancillary assumptions that help make the empirical exercise feasible. The issue is that these assumptions can be taken onboard later, perhaps inadvertently, as theoretical commitments. Since these originate from small-scale, low-complexity problems, they are likely to be problematic for the reasons we mentioned earlier (§“The Challenge of Small-Scale Domains”). Upgrading these local problem assumptions to the level of theory usually sidesteps some theoretical constraints at the cost of introducing other theoretical problems. Hence, these will have to be surfaced later on through theoretical problem-finding. A focus on empirical problem-solving thereafter cannot help, as it will select models based on their hidden ancillary assumptions (Navarro, 2019). The following case study illustrates these and other issues.

In communication, people often need to resolve misunderstandings or ambiguous messages (Dingemanse et al., 2015; Fusaroli et al., 2017; Healey et al., 2018). In most computational models that describe how conventions are formed in communication, unambiguous explicit feedback is incorporated as a cognitive crutch to assist in reaching mutual understanding. However, in real life, people do not always need, or even have access to, such explicit feedback. Yet, they communicate successfully. Consider, for example, a phone conversation. Here, it is not possible to physically point at any object one wishes to discuss. Nevertheless, in such scenarios, people still manage to communicate about these objects. This is a design feature of language known as *displacement*; meaning, we can refer to things not in the here and now (Hockett, 1960). From the point of view of explaining communication, the availability and use of feedback as a necessary component is therefore an inappropriate theoretical/modeling assumption.

Having uncovered the inadequacy of this assumption, van de Braak et al. (2021) conducted computational modeling work to investigate how reaching such mutual understanding could be done without it. Surprisingly, simulations showed that removing the assumption results in models performing at chance level, even when sophisticated reasoning is provided to compensate for the lack of explicit feedback. This resulted in a standoff between the full generality of the phenomenon as conceptualized and the necessary ancillary assumptions for the models to “work” empirically. As the models are (supposed to be) tied to proposed explanations for the phenomenon, this naturally leads to the conclusion that, as they stand, these explanations are insufficient.

This is an example of a *cognitive scope violation* (defined back in §“Plausibility Constraints as Theoretical Problems”). The assumption at the root of this was incorporated into the theory after an unwarranted focus on a specific set of empirical results rather than real-world theoretical problems (as discovered in van de Braak et al. n.d.). It was included because otherwise the model would not have been able to “solve” the problematizing datasets at hand (the empirical problems). In other words, it is required for the model to “work” as intended in the smaller context of the experimental setup. Real-world cognition does not need the crutch.

There is an unfortunate link between the so-called empirical problems at hand and the theoretical problems at play. An atheoretical ancillary assumption is made with the goal of improving the performance of the model on a given dataset. This is one clear case wherein such an ancillary assumption undermines eventual cognitive explanations. Here, as we have been arguing to be the case more generally, a narrow view of cognitive science as empirical problem-solving initially resulted in impoverishing our theoretical understanding of cognition. Theoretical problem-finding later restored the latent cognitive-theoretic problems facing researchers in this domain and allowed them to assert the status of available explanations and to stand on firmer ground.

This theoretical problem-finding work led the authors to the following conclusions: *“This shows that state-of-the-art computational explanations have difficulty explaining how people solve the puzzle of underdetermination, and that doing so will require a fundamental leap forward.”* (van de Braak et al., 2021). This change to the theory *must* be fundamental if we are to reach a plausible explanation. The reason is that conjecturing an alternative explanation that does not lean on the same flawed foundation requires a creative leap away from, not merely an amendment of, the previous explanation. Theoretical problem-finding is integral to indicate clear future paths for theoretical research. Naturally, then, this meta-theoretical insight can be and should be allowed to detach from a “solution” to the problem. We return to this shortly before closing.

### Exploring the Boundaries of Plausible Theories

The reader may wonder whether theoretical problem-finding extends only as far as pointing out issues with existing theories. We believe its reach is far greater and more forward-looking. To illustrate this point, consider the following case study (see van Rooij et al. 2011, for full details).

In the field of human communication, explaining how humans communicate successfully at real-world speed is a challenge (Levinson, 1995). Van Rooij et al. (2011) propose a methodology to perform parameterized complexity analyses (Downey & Fellows, 2013; Wareham, 1996) to identify possible real-world constraints under which a model of communication can be tractable. These analyses characterize the boundary separating plausible from implausible theories under the constraint of tractability. After establishing intractability results for an unconstrained model, the authors state the following: *“Importantly, our analyses do not stop at the intractability results [...]. On the contrary, we view such results as merely the fruitful starting point of rigorous analyses of the sources of complexity in human communication.”* (van Rooij et al., 2011). They then go on to perform such analyses, which led to identifying a set of parameter restrictions which, if appropriate to characterize real-world conditions, would satisfy plausibly the tractability constraint on the theory. These parameter restrictions can be translated to real-world situational constraints on the phenomenon that is being modeled. Hence, a natural next step is to check, empirically (i.e., via observation and experiment), whether these restrictions hold and if they fulfill the expected roles. For instance, these formal results characterizing what is in principle possible can inform cognitive neuroscience studies investigating whether and how humans exploit these restrictions. Importantly, without the findings from these formal analyses, we would not know where to look.

This brief example illustrates how the integration of theoretical problem-finding into our research practices can inform future theory development for both theoretical and empirical research. The boundaries discovered in this way can be useful in further theoretical research, informing further steps for the sculpting of theory (Blokpoel, 2018; van Rooij & Blokpoel, 2020). They can also be used in empirical research, as they help determine which experiments might be theoretically meaningful.

## Problem-Finding Without Problem-Solving

*“I do not offer answers to these questions, but hope to highlight the reasons why psychological researchers cannot avoid asking them”* (Navarro, 2019)

Finally, let us explore whether it is sensible to expect a “problematizing dataset” whenever theoretical problems are raised. This parallels the question whether problem-finding without problem-solving is desirable and, in particular, whether theoretical problem-finding in the absence of empirical problem-solving is possible and necessary.

In some cases, seeking a problematizing set of observations may be a sensible *follow-up* (i.e., not an immediate requirement) to theoretical problem-finding, but we argue it is not a reasonable expectation in general. Gathering such a problematizing dataset can be either (i) too costly compared to theoretical work^10^; (ii) practically infeasible in certain cases (either in the near future or in general); (iii) too hard because it requires separating relevant from irrelevant aspects of the problem—a task which cannot be efficiently proceduralized (Kwisthout, 2012); or (iv) simply impossible. That is, even if we could have a problematizing dataset in principle, which is by no means obvious in any given case, it would not seem sensible to immediately expect one.

First, the empirical research necessary to generate such a dataset may be too costly in terms of time or resources. Taking tractability as an example: building a dataset of real-world complexity in all relevant dimensions to assess whether a theory scales beyond toy domains is not guaranteed to be possible. For instance, when it comes to cognitive processes that take place over a long time scale, such as learning, development, or even (cultural) evolution, datasets of ecological scale are likely infeasible to gather through observational or experimental work (either in the near future or at all). Second, interventions of interest on processes such as cognitive development or evolution are in most cases unethical. In such cases, research often takes the form of comparatively small-scale, low-complexity experiments that rely on non-trivial assumptions, such as assuming continuity between the cognition of modern-day human participants and the cognition of our hominin ancestors (such as in the iterated learning experiments investigating language evolution discussed in Section §“The Challenge of Small-Scale Domains”) (Woensdregt et al., in press). Furthermore, such small-scale experiments require being combined with a tractable computational model (and thus tractability analyses; §“Plausibility Constraints as Theoretical Problems”) in order to link between the low-complexity data and the ecological-scale implications of the theory (see §“The Challenge of Small-Scale Domains”). Third, building a problematizing dataset can be intrinsically hard because it requires a subset of *relevant* dimensions of the empirical problem to be carved out among all those that could in principle be relevant (Kwisthout, 2012).

Therefore, a reframing of theoretical problems in terms of problematizing datasets cannot and should not be expected to follow directly from theoretical problem-finding exercises. To conclude, theoretical problem-finding without problem-solving is possible and necessary.

## Outroduction

We have come a long way from an empirical problem-solving frame for Cognitive Science to one that integrates theoretical problem-finding. This journey casts doubts on the idea that cognitive capacities are somehow directly intelligible to cognitive scientists through (solving) empirical problems, narrowly construed. As cognitive scientists and meta-scientists, we can instead redirect our gaze towards the possibility that cognitive science involves actively carving out (theoretically problematic) empirical phenomena and constructing *cognitive theories* that are intelligible to us. Here, we have begun to bring *theoretical problem-finding* out of obscurity as a core research activity towards such purposes. Cognitive Science needs (theoretical) problem-finding as much as (empirical) problem-solving.

## Data Availability

Not applicable.

## Footnotes

1 *Retrodiction* sometimes called *postdiction*, refers to the accounting of data gathered in conditions already observed prior to the relevant theoretical development. This is in contrast to *prediction*, where novel data is accounted for.

2 Note here, in this narrow sense of *solving* the lack of reference to criteria for when observations could be considered *explained*.

3 A more fine-grained distinction can be drawn between internal and external non-empirical problems. Internal problems involve inconsistencies or contradictions within a given theory. External problems involve incompatibility with independent theories or principles. (For illustrative purposes, we focus on this latter type throughout.)

4 Indeed, in our view, the distinction between empirical and theoretical problems can itself be misleading if taken at face value (we illustrate in section §“Integrating Problem-Solving and Problem-Finding”).

5 This new theoretical development is often taken up by problem finders themselves.

6 We address the question of whether it is sensible to require a “problematizing dataset” for every theoretical problem in §“Problem-Finding Without Problem-Solving”.

7 These lenses are all correct, or truthful, as far as we are concerned. Our purpose here is not to provide the most veridical frame (whatever that may be) but to restore a lens that we believe is lacking or whose existence has been negated by misconceptions. Hence, although we focus here on the theoretical problem-finding aspect, our overarching hope is to see a diversity of lenses restored.

8 See van Rooij et al. (2023) for a related discussion involving machine learning artifacts as cognitive models.

9 Specifically, this is the case for *iterated Bayesian language learning* models that use signal-meaning mappings (i.e., a lexicon) as their model of language.

10 Note we often confuse the cost of research activities with the scientific value of their possible outcomes.
